# Pathophysiological significance of increased α-synuclein deposition in sympathetic nerves in Parkinson’s disease: a post-mortem observational study

**DOI:** 10.1186/s40035-022-00289-y

**Published:** 2022-03-08

**Authors:** Risa Isonaka, Patti Sullivan, David S. Goldstein

**Affiliations:** Autonomic Medicine Section, Clinical Neurosciences Program, Division of Intramural Research, National Institute of Neurological Disorders and Stroke, National Institutes of Health, Bethesda, MD 20892 USA

**Keywords:** Synuclein, Tyrosine hydroxylase, Norepinephrine, Immunofluorescence, Parkinson, Post-mortem

## Abstract

**Background:**

Parkinson’s disease (PD) is characterized by intra-neuronal deposition of the protein α-synuclein (α-syn) and by deficiencies of the catecholamines dopamine and norepinephrine (NE) in the brain and heart. Accumulation of α-syn in sympathetic noradrenergic nerves may provide a useful PD biomarker; however, whether α-syn buildup is pathophysiological has been unclear. If it were, one would expect associations of intra-neuronal α-syn deposition with catecholaminergic denervation and with decreased NE contents in the same samples.

**Methods:**

We assayed immunoreactive α-syn and tyrosine hydroxylase (TH, a marker of catecholaminergic innervation) concurrently with catecholamines in coded post-mortem scalp skin, submandibular gland (SMG), and apical left ventricular myocardial tissue samples from 14 patients with autopsy-proven PD and 12 age-matched control subjects who did not have a neurodegenerative disease.

**Results:**

The PD group had increased α-syn in sympathetic noradrenergically innervated arrector pili muscles (5.7 times control, *P* < 0.0001), SMG (35 times control, *P* = 0.0011), and myocardium (11 times control, *P* = 0.0011). Myocardial TH in the PD group was decreased by 65% compared to the control group (*P* = 0.0008), whereas the groups did not differ in TH in either arrector pili muscles or SMG. Similarly, myocardial NE was decreased by 92% in the PD group (*P* < 0.0001), but the groups did not differ in NE in either scalp skin or SMG.

**Conclusions:**

PD entails increased α-syn in skin, SMG, and myocardial tissues. In skin and SMG, augmented α-syn deposition in sympathetic nerves does not seem to be pathogenic. The pathophysiological significance of intra-neuronal α-syn deposition appears to be organ-selective and prominent in the heart.

**Supplementary Information:**

The online version contains supplementary material available at 10.1186/s40035-022-00289-y.

## Background

Lewy bodies containing the protein alpha-synuclein (α-syn) in the cytoplasm of brainstem catecholaminergic neurons are a characteristic feature of Parkinson’s disease (PD) [1]. α-Syn is also found in nerve processes (Lewy neurites) [2].

PD entails Lewy neurites both inside the brain and in several extra-cranial organs [3–5] including potentially biopsiable skin [6], submandibular gland (SMG) [7], and gastrointestinal tract [8]. Post-mortem studies have noted α-syn in sympathetic ganglionic neurons [9–11], epicardial nerves [12], and left ventricular myocardium [5, 13] in PD. With rare exception the assays have been semi-quantitative [11].

Increased α-syn deposition in tissues does not necessarily mean that the buildup is pathogenic. One way to assess the pathophysiological significance of such deposition is by measuring immunoreactive tyrosine hydroxylase (TH). TH is the rate-limiting enzyme in catecholamine biosynthesis [14], and immunoreactive TH is a marker of catecholaminergic neurons [15]. Several publications have noted decreased immunoreactive TH in sympathetic neurons supplying the heart in PD. Low levels of immunoreactive TH have been observed in sympathetic ganglia [9], epicardial nerve fascicles [16], and myocardium [13]. Estimates of the extent of decrease in cardiac immunoreactive TH in PD have varied considerably, from about 50% to almost 100% [12, 13, 17, 18]. The assays for immunoreactive TH in these studies have been subjective or semi-quantitative.

It is well known that depletion of the catecholamine dopamine (DA) in the nigrostriatal system characterizes and likely produces the movement disorder that defines PD. Outside the brain, PD is associated with severe deficiency of the closely related catecholamine norepinephrine (NE) in the left ventricular myocardium [19, 20]. NE is the main neurotransmitter of the sympathetic nervous system in regulation of the circulation, and NE content is the gold standard for assessing sympathetic noradrenergic deficiency. The finding of myocardial NE depletion in PD implies a substantial cardiac sympathetic noradrenergic lesion; however, this lesion might be from denervation, from decreased ability to synthesize, store, or recycle NE, or some combination of denervation with dysfunctions in residual nerves.

The purpose of this post-mortem observational study was to measure α-syn, TH, and catecholamine contents concurrently in the same tissue samples from scalp skin, SMG, and left ventricular apical myocardium from patients with autopsy-proven PD. Obtaining simultaneous microscopic immunofluorescence and catecholamine neurochemical information enables evaluation of the pathophysiological significance of α-syn deposition in sympathetic nerves. If intra-neuronal α-syn accumulation were pathogenic, then one would expect associations of α-syn buildup with decreased immunoreactive TH and with decreased NE contents in the same samples. For comparison purposes, data were obtained from an age-matched group of control subjects who did not have a neurodegenerative disease.

## Materials and methods

### Human subject research

The NIH Office of Human Subjects Research Protections determined that this post-mortem study was exempt from the requirement of Institutional Review Board approval. In all cases, the tissues were harvested for research purposes after consent of the next of kin.

### Study design

This was an observational, retrospective, cross-sectional study of coded post-mortem samples from patients with autopsy-proven PD and from control subjects with similar age and sex distribution and no history of a neurodegenerative disease. The numbers of subjects in the two groups that would be required to test the main hypothesis of the study were determined based on previous publications about myocardial NE in Lewy body diseases [20, 21]. According to a power analysis, for 2 independent study groups, a dichotomous primary endpoint, alpha 0.05, and power 80%, a total of 10 data points per group would be needed.

### Tissue samples

Scalp skin, SMG, and apical myocardial tissue samples were harvested at autopsy from the same patients. Samples from 11 PD patients and 12 control subjects were obtained from the Banner Sun Health Research Institute (Sun City, AZ) under an approved Material Transfer Agreement. All the samples were frozen upon tissue harvesting and kept frozen until shipped to our laboratory. The samples were not fixed. Banner did not provide clinical data about the PD patients or control subjects. There were 3 non-Banner PD patients, including 1 autopsied at the NIH and 2 at outside institutions, for a total of 14 PD patients.

### Immunofluorescence microscopy

The samples were embedded in optimum cutting temperature compound and sliced into 8–10 μm thick sections (Histoserv, Germantown, MD). The immunofluorescence staining procedures for visualizing α-syn and TH followed our previously published methodology [11]. Briefly, each examined slide had a coded, stock positive control slice from a patient with a Lewy body disease and a negative control slice. For each tissue, images were obtained using the same microscopic laser settings.

Fluorescence of the staining for α-syn and TH was expressed in absolute terms as well as in terms of ratios to alpha-smooth muscle actin (SMA) in scalp skin and SMG or to cardiac troponin T (cTnT) in myocardium. The primary antibodies used were mouse IgG_1_ monoclonal anti-total α-syn (1:1000; sc-69977; Santa Cruz Biotechnology, Santa Cruz, CA), rabbit anti-TH (1:1000; P40101; Pel-Freez Biologicals, Rogers, AR), mouse IgG_2a_ monoclonal anti-SMA (1:400; ab7817; Santa Cruz Biotechnology), and mouse IgG_2a_ monoclonal anti-human cTnT (1:250; MAB1874; Pel-Freez Biologicals). The primary immunoreactions were visualized using the following secondary antibodies: Alexa 488-conjugated anti-mouse IgG_1_, Alexa 555-conjugated anti-rabbit, and Alexa 647-conjugated anti-mouse IgG_2a_ (Thermo Scientific, Inc, Rockford, IL). Coverslips were mounted on slides with ProLong Gold antifade reagent (Thermo Scientific), and the slides examined by immunofluorescence confocal microscopy using a Zeiss LSM 880 confocal laser scanning microscope (Carl Zeiss, Oberkochen, Germany). Image sizes were 1024 × 1024 pixels.

### Catechol assays

Catechol assays were performed on the same samples as those analyzed by immunofluorescence. The tissue samples were split for immunofluorescence and neurochemistry. Tissue specimens for catechol assays were kept frozen at − 80 °C until thawed for assay, which was by batch alumina extraction followed by liquid chromatography with series electrochemical detection, as described previously [19]. Data were obtained for the catecholamines NE and DA as well as for 3,4-dihydroxyphenylglycol (DHPG), which is the main intra-neuronal metabolite of NE, and for 3,4-dihydroxyphenylacetic acid (DOPAC), which is the main intra-neuronal metabolite of DA.

### Data analysis and statistics

For each scalp skin sample, all arrector pili, blood vessel, and sweat gland structures in the tissue slice were analyzed. In scalp skin, the average fluorescence intensities of α-syn, TH, and SMA in regions of interest in arrector pili muscles, blood vessels, and sweat glands in scalp skin were quantified using Fiji software with background subtraction [11]. Ratios of α-syn/SMA, TH/SMA, and α-syn/TH were calculated.

In SMG and myocardium, integrated absolute intensities of α-syn, TH, and SMA (in SMG) and cTnT (in myocardium) were measured. In images from SMG, α-syn/SMA, TH/SMA, and α-syn/TH ratios were calculated, and in images from myocardium, α-syn/cTnT, TH/cTnT, and α-syn/TH ratios were calculated.

For a description of the power analysis please see the “Study design” section. Because of skewed distributions of individual values for some analytes, the non-parametric Mann–Whitney U test was used to compare the PD *vs* control groups for immunoreactive α-syn and TH and for tissue contents of catechols. Data for the two groups were expressed as means ± SEM. A *P* value less than 0.05 defined statistical significance.

### Avoidance of biases

The immunofluorescence staining, microscopy and image analysis and the neurochemical assays were done on coded samples by different personnel who were blinded to the diagnostic group until the data were tabulated.

## Results

### Subject groups

The PD group consisted of 10 males and 4 females with a mean age of 80 ± 2 years (range 63–91 years). The control group consisted of 7 males and 5 females with a mean age of 82 ± 2 years (range 74–93 years) (Additional file 1). The control subjects did not have a neurodegenerative disease or brainstem Lewy bodies. In all the PD patients the diagnosis was confirmed by standard post-mortem neuropathology.

All the post-mortem intervals were less than 24 h, except for 1 control subject who had a post-mortem interval of 72 h. This exception was not discovered until after the assays had been done and the results tabulated. The data from this subject did not stand out from those of others in the control group and were included in the analyses.

### Missing data

Datasets were complete for α-syn, TH, and NE for all three tissues, scalp skin, SMG, and myocardium. Some other neurochemical data were missing, as follows. In the PD group, there were no data about DA for 6 myocardial samples, due to interfering co-chromatographic peaks. There were 6 myocardial samples without DHPG data (1 control, 5 PD), mainly due also to interfering peaks. Because of the number of missing data points for myocardial DA and DHPG, statistical testing was not done comparing myocardial tissue levels of these catechols in the PD *vs* control groups, and only descriptive statistics are reported. There were no data for skin tissue DHPG in 2 PD patients and for skin tissue DOPAC in 1 control subject due to interfering peaks. In another PD patient, no arrector pili muscles were found in the skin sample, and in 1 control subject and 3 PD patients no sweat glands were found in the skin samples.

### α-Syn deposition in scalp skin, SMG, and myocardium

**Fig. 1:**
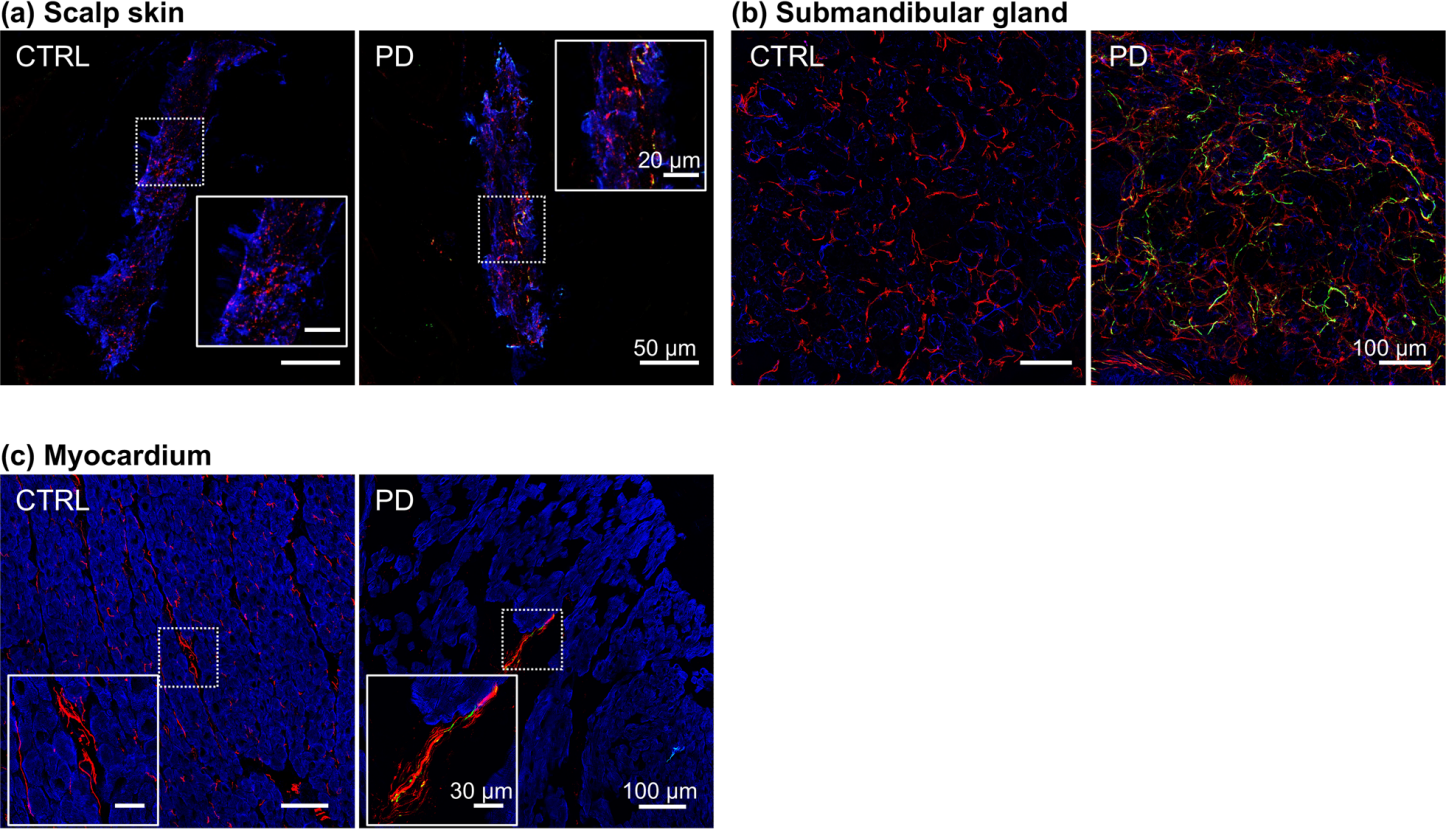
Representative immunofluorescence confocal microscopic images from a control subject (CTRL) and a PD patient. Immunoreactive tyrosine hydroxylase (TH) indicates sympathetic noradrenergic innervation in an arrector pili muscle in scalp skin tissue (**a**), in submandibular gland (SMG, (**b**)), and in myocardium (**c**). Green shows α-synuclein (α-syn), and red shows TH. Blue shows smooth muscle actin (arrector pili muscles and SMG) or cardiac troponin T (cTnT, myocardium). The regions surrounded by white squares are magnified in the inserted images. Note increased α-syn deposition in arrector pili muscle, SMG, and myocardium in the PD patient but not in the CTRL subject

**Fig. 2:**
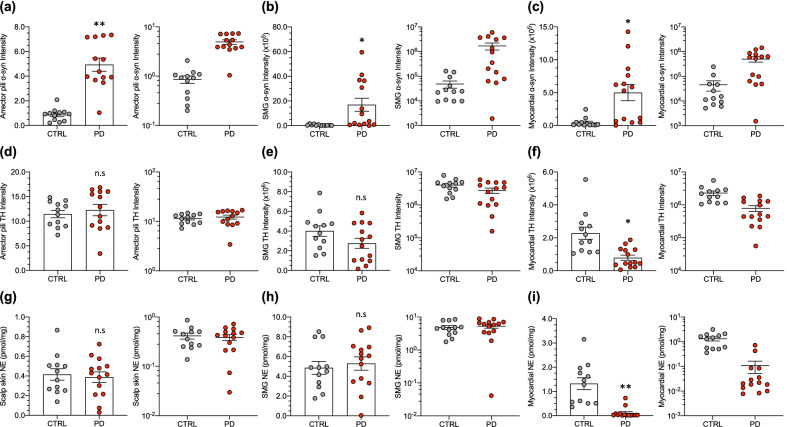
Individual values and means ± SEM for immunoreactive signal intensities of α-synuclein (α-syn) and tyrosine hydroxylase (TH) and for concentrations of norepinephrine (NE, pmol/mg wet weight) in control (CTRL) subjects (gray) and PD patients (red). In each panel, linear (left) and logarithmic scales (right) are used. (**a**–**c**) α-Syn intensities in arrector pili muscles, submandibular gland (SMG), and myocardium. (**d**–**f**) TH signal intensities in these tissues, and (**g**–**i**) NE concentrations. Note α-syn intensities were increased in all 3 tissues in the PD group compared to the CTRL group (**a**–**c**). Myocardial TH intensity was moderately decreased (**f**), and myocardial NE was substantially decreased (**i**) in the PD group. The groups did not differ in contents of TH or NE in skin (**d**, **g**) or SMG (**e**, **h**). (*) *P* < 0.005, (**)*P* < 0.0001 for PD *vs.* CTRL. *n.s.* non-significant difference

Compared to the control group, the PD group had increased α-syn deposition in scalp skin (arrector pili muscles, blood vessels, and sweat glands), SMG, and myocardium. Illustrative images are shown in Fig. 1 and individual and summary data in Fig. 2. In the PD patient, α-syn, TH, and α-syn-TH colocalization were visualized, whereas in the control subject there was little if any visible α-syn or α-syn-TH colocalization (Fig. 1).

Regarding sympathetic noradrenergically innervated constituents in scalp skin, in the PD group, α-syn signal intensities in arrector pili muscles averaged 5.7 times those in the control group (*P* < 0.0001; Fig. 2a). Increased α-syn deposition was observed in the PD group regardless of adjustment for SMA in the regions of interest (Table 1). In the PD group, α-syn signal intensities in blood vessels averaged 3.9 times those in the control group, and the increased α-syn deposition was observed regardless of adjustment for SMA. In sweat glands, the mean α-syn signal intensity in the PD group was 3.9 times that in the control group, and the α-syn deposition was increased regardless of adjustment for SMA.

**Table 1:** Immunofluorescence signal intensities and related ratios in control (CTRL) and PD groups

Parameter	CTRL	PD	*P* value
*Scalp skin*			
Arrector pili muscles			
α-Syn intensity	0.861 ± 0.149 (12)	4.938 ± 0.533 (13)	< 0.0001
α-Syn/SMA	0.025 ± 0.004 (12)	0.156 ± 0.012 (13)	< 0.0001
Blood vessels			
α-Syn intensity	1.187 ± 0.241 (12)	4.571 ± 0.482 (14)	< 0.0001
α-Syn/SMA	0.057 ± 0.010 (12)	0.213 ± 0.015 (14)	< 0.0001
Sweat glands			
α-Syn intensity	1.112 ± 0.302 (11)	4.324 ± 0.661 (11)	< 0.0001
α-Syn/SMA	0.075 ± 0.017 (11)	0.246 ± 0.028 (11)	< 0.0001
*SMG*			
α-Syn intensity (× 10^5^)	0.486 ± 0.159 (12)	16.932 ± 5.322 (14)	0.0011
α-Syn/SMA	0.007 ± 0.002 (12)	0.123 ± 0.034 (14)	0.0003
*Myocardium*			
α-Syn intensity (× 10^5^)	0.455 ± 0.202 (12)	4.999 ± 1.231 (14)	0.0011
α-Syn/cTnT	0.002 ± 0.001 (12)	0.020 ± 0.005 (14)	0.0007
*Scalp skin*			
Arrector pili muscles			
TH intensity	11.438 ± 0.737 (12)	12.256 ± 1.149 (13)	0.3760
TH/SMA	0.332 ± 0.023 (12)	0.406 ± 0.039 (13)	0.2051
α-Syn/TH	0.075 ± 0.012 (12)	0.407 ± 0.028 (13)	< 0.0001
Blood vessels			
TH intensity	6.084 ± 0.606 (12)	7.250 ± 0.676 (14)	0.2682
TH/SMA	0.338 ± 0.057 (12)	0.344 ± 0.026 (14)	0.4319
α-Syn/TH	0.225 ± 0.049 (12)	0.647 ± 0.044 (14)	< 0.0001
Sweat glands			
TH intensity	5.688 ± 1.011 (11)	6.842 ± 0.821 (11)	0.3000
TH/SMA	0.405 ± 0.082 (11)	0.418 ± 0.057 (11)	0.6522
α-Syn/TH	0.189 ± 0.031 (11)	0.735 ± 0.141 (11)	< 0.0001
Coloc. Index	1.076 ± 0.196 (12)	2.278 ± 0.099 (14)	< 0.0001
*SMG*			
TH intensity (× 10^6^)	4.010 ± 0.531 (12)	2.756 ± 0.509 (14)	0.1448
TH/SMA	0.555 ± 0.084 (12)	0.317 ± 0.048 (14)	0.0407
α-Syn/TH	0.017 ± 0.007 (12)	0.537 ± 0.198 (14)	< 0.0001
Coloc. Index	0.885 ± 0.263 (12)	2.612 ± 0.331 (14)	0.0003
*Myocardium*			
TH intensity (× 10^6^)	2.280 ± 0.373 (12)	0.789 ± 0.157 (14)	0.0008
TH/cTnT	0.080 ± 0.011 (12)	0.031 ± 0.006 (14)	0.0005
α-Syn/TH	0.024 ± 0.011 (12)	0.810 ± 0.208 (14)	< 0.0001
Coloc. Index	0.669 ± 0.219 (12)	0.909 ± 0.263 (14)	0.4700

Mean ± SEM. Numbers in parentheses are numbers of data points. Listed *P* values are for the non-parametric Mann–Whitney test

*α-Syn* = α-synuclein, *Coloc. Index* = α-synuclein-tyrosine hydroxylase colocalization index, *cTnT* = cardiac troponin T, *SMA =* smooth muscle actin, *SMG* = submandibular gland, *TH* = tyrosine hydroxylase

In SMG specimens, α-syn signal intensities in the PD group averaged 35 times those in the control group (*P* = 0.0011; Fig. 2b). The increased α-syn deposition was observed regardless of adjustment for SMA (Table 1). In myocardial specimens, α-syn signal intensities in the PD group averaged 11 times control (*P* = 0.0011; Fig. 2c). The α-syn deposition was increased regardless of adjustment for cTnT (Table 1).

### Immunoreactive TH in scalp skin, SMG, and myocardium

For all 3 skin constituents, the PD group did not differ from the control group in TH signal intensities, regardless of adjustment for SMA (Table 1; Fig. 2d). The PD and control groups did not differ in absolute TH signal intensities in SMG (Table 1; Fig. 2e); however, in SMG the PD group had a lower mean TH/SMA ratio (*P* = 0.0407).

In myocardium, the tissue TH signal intensities in the PD group were decreased from those in the control group, by a mean of 65% (*P* = 0.0008; Fig. 2f). The decreases in TH signal intensities in the PD group were significant regardless of adjustment for cTnT (Table 1). One of the control subjects had a high value for myocardial immunoreactive TH (Fig. 2f). After exclusion of this data point, myocardial mean TH in the PD group remained significantly decreased from the mean TH in the control group (*P* = 0.0018).

After adjustment of α-syn signal intensities for TH in the same samples, α-syn/TH ratios in arrector pili muscles, blood vessels, and sweat glands in the PD group averaged 5.4, 2.9, and 3.9 times those in the control group (*P* < 0.0001 each, Table 1). In the PD group, the mean α-syn/TH ratio in SMG was 32 times that in the control group (*P* < 0.0001). In myocardium, the mean α-syn/TH ratio in the PD group was 34 times that in the control group (*P* < 0.0001). The mean values for α-syn-TH colocalization indices in scalp skin and SMG were higher in the PD group than in the control group (*P* < 0.0001 and *P* = 0.0003; Table 1). In contrast, in myocardium the mean α-syn-TH colocalization index in the PD group did not differ from that in the control group.

### Catecholamines in scalp skin, SMG, and myocardium

The PD group did not differ from the control group in the mean NE contents for either skin or SMG samples (Fig. 2g, h; Table 2). In contrast, in the same patients, the mean myocardial concentration of NE was drastically decreased from that in the control group, by 92% (*P* < 0.0001; Fig. 2i). The control subject with the lowest myocardial NE content in the group also had the highest myocardial α-syn signal intensity.

**Table 2:** Tissue catechol contents in control (CTRL) and PD groups

Parameter	CTRL	PD	*P* value
*Scalp skin*			
NE (pmol/mg)	0.415 ± 0.058 (12)	0.387 ± 0.054 (14)	0.8201
DA (pmol/mg)	0.026 ± 0.010 (12)	0.031 ± 0.009 (9)	*
DHPG (pmol/mg)	0.016 ± 0.005 (12)	0.007 ± 0.001 (12)	0.0501
DOPAC (pmol/mg)	0.017 ± 0.003 (11)	0.011 ± 0.002 (14)	0.1492
*SMG*			
NE (pmol/mg)	4.851 ± 0.656 (12)	5.296 ± 0.674 (14)	0.4624
DA (pmol/mg)	0.251 ± 0.100 (12)	0.254 ± 0.071 (14)	0.8596
DHPG (pmol/mg)	0.387 ± 0.068 (12)	0.495 ± 0.117 (14)	0.5952
DOPAC (pmol/mg)	0.060 ± 0.019 (12)	0.096 ± 0.030 (14)	0.6308
*Myocardium*			
NE (pmol/mg)	1.322 ± 0.246 (12)	0.108 ± 0.056 (14)	< 0.0001
DA (pmol/mg)	0.138 ± 0.055 (12)	0.006 ± 0.003 (8)	*
DHPG (pmol/mg)	0.036 ± 0.011 (11)	0.004 ± 0.001 (9)	*
DOPAC (pmol/mg)	0.032 ± 0.011 (12)	0.006 ± 0.001 (14)	0.0056

Mean ± SEM. Numbers in parentheses are numbers of data points. Listed *P* values are for the non-parametric Mann–Whitney test. (*) indicates statistical testing not done, due to missing data points

*DA* = dopamine, *DHPG* = 3,4-dihydroxyphenylglycol, *DOPAC* = 3,4-dihydroxyphenylacetic acid, *NE* = norepinephrine, *SMG* = submandibular gland

In the PD group, the myocardial DA averaged 4% that in the control group (Table 2). The mean tissue content of DHPG was 11% that in the control group. The mean myocardial content of DOPAC in the PD group averaged 18% that in the control group (*P* = 0.0056; Table 2).

### Inter-relationships among immunoreactive α-syn, TH, and NE in myocardium

**Fig. 3:**
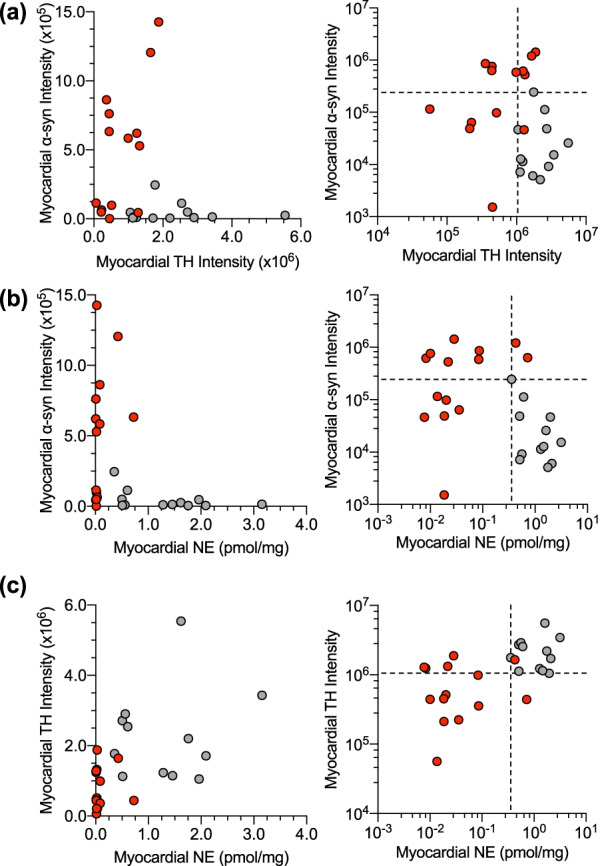
Individual values for myocardial α-syn or TH signal intensities as functions of the apical myocardial concentration of norepinephrine (NE, pmol/mg wet weight) in control subjects (CTRL, gray) and in PD patients (red). Data are displayed using linear and logarithmic scales. Panel (**a**) shows data for α-syn *vs. *TH, (**b**) α-syn *vs.* NE, and **c** TH *vs.* NE. Dashed lines show upper and lower limits of values in the CTRL group. Note complex inter-relationships among individual values for α-syn, TH, and NE in the PD group

Among the 14 PD patients, 8 had myocardial α-syn signal intensities that were above the control range (Fig. 3a); 4 had both α-syn above the control range and TH below the control range, and 5 had low TH without increased α-syn. Twelve of the 14 PD patients had myocardial NE contents below the control range (Fig. 3b); among these, 6 had α-syn above the control range, and 6 did not. Twelve of the 14 PD patients had myocardial NE below the control range; of the 12, 8 had TH below the control range and 4 did not (Fig. 3c).

## Discussion

Concurrent measurements of tissue α-syn, TH, and catecholamines have not been reported previously in PD. As explained below, combining immunofluorescence microscopic with catecholamine neurochemical data enabled insights into the pathophysiological significance of α-syn deposition in sympathetic nerves in PD.

If increased α-syn deposition were pathogenic, then one would expect intra-neuronal α-syn buildup to be associated with decreased immunoreactive TH and with decreased NE contents in the same samples. We found increased intra-neuronal α-syn deposition in all three tissues, but we did not find decreased TH or NE content in skin or SMG. Instead, decreased TH and NE contents were observed only in the myocardial tissue. Therefore, although intra-neuronal α-syn deposition in biopsiable skin or SMG might provide an in vivo biomarker of PD, our results suggest that in sympathetic nerves in these tissues increased α-syn deposition does not seem to be pathogenic. On the other hand, intra-neuronal α-syn deposition may be pathogenic in myocardium.

### Immunoreactive α-syn is increased in scalp skin, SMG, and myocardium in PD

The present results about α-syn deposition in skin, SMG, and myocardium in PD agree with those in previous reports [6, 7] and confirm that PD entails increased α-syn deposition not only in the brain but also in the periphery, consistent with a generalized or multi-organ disease process rather than with a disease that is confined to the brain. The present data provide important extensions, in that quantitative methodology was used, enabling calculation of α-syn-TH colocalization indices and comparisons of the extents of decrease in TH and catecholamine contents in the same samples.

### Immunoreactive TH is decreased in myocardium but not in scalp skin or SMG in PD

In PD, post-mortem immunoreactive TH has been reported to be decreased in the myocardium [18] based on semi-quantitative assay methodology and has not yet been measured by any technique in skin or SMG. In the present study, application of quantitative methodology revealed substantially decreased immunoreactive TH in apical myocardial samples from PD patients, with or without adjustment for cTnT. The PD and control groups did not differ in immunoreactive TH in scalp skin or SMG. Thus, decreased immunoreactive TH is not a generalized finding in PD and is prominent in the heart.

Sympathetic innervation to the scalp skin, SMG, and heart passes through the thoracic spinal cord and ganglia. The distances traversed by the post-ganglionic fibers would be expected to be about the same for the three target organs. Accordingly, the observed differences among these organs in the extents of decrease in immunoreactive TH cannot be explained by length-dependent sympathetic denervation.

### Catecholamine deficiency in myocardium but not in scalp skin or SMG in PD

Measuring tissue contents of catecholamines is the benchmark for identifying and quantifying sympathetic noradrenergic deficiency. In the present study, we observed the same general pattern of results by catecholamine neurochemistry as by immunoreactive TH, with evidence for profound catecholamine deficiency in the heart but not in the skin or SMG in PD. Myocardial contents of NE and DA in the PD group averaged 8% and 4% of control, whereas the PD and control groups did not differ in mean values for either catecholamine in scalp skin or SMG.

### Complex inter-relationships among myocardial α-syn, TH, and NE in PD

Scatter plots relating individual values for α-syn, TH, and NE in PD were complex and did not fit with a simple pathophysiological picture. Low NE was almost universal, but α-syn buildup and decreased TH were not. This inconsistency may be explained as follows. Measuring the amount of α-syn in the tissue does not address where the α-syn is localized within the tissue. If in the post-mortem PD tissue, α-syn were deposited only extra-neuronally, such as in the form of α-syn fibrils dispersed in the myocardium, then if there were a decrease in the number of nerve fibers, there would still be α-syn signal in the tissue. In this situation, TH would be low, NE would be low, but α-syn would not be low. The α-syn-TH colocalization index would be zero. In the extreme opposite situation, if the α-syn in the PD tissue were deposited only within the sympathetic noradrenergic nerves, then if there were a decrease in the number of nerve fibers, there would be a corresponding decrease in the amount of α-syn signal in the tissue. In this situation, TH would be low, NE would be low, α-syn would be low, and the α-syn-TH colocalization index would also be low. In an intermediate situation, if the extent of decrease in nerves were mild, and if α-syn were deposited within the residual nerves, then TH would be mildly decreased, NE would be mildly decreased, α-syn would be increased, and the α-syn-TH colocalization index would be higher than control. These different scenarios may help explain why inter-relationships among individual values for myocardial α-syn, TH, and NE were complex and why the mean α-syn-TH colocalization index did not differ between the PD and control groups.

The post-mortem format of research we used obviated drawing inferences about whether α-syn buildup in myocardial sympathetic nerves is pathogenic. For instance, there could be a common upstream determinant of both α-syn buildup and decreased TH.

There is in vivo evidence that α-syn deposition can be pathogenic in cardiac sympathetic nerves. In patients with PD from genetic synucleinopathy, myocardial ^18^F-DA-derived radioactivity, an in vivo index of cardiac NE content  [21], is decreased [22, 23], which supports the view that synucleinopathy can be pathogenic in cardiac sympathetic nerves; however, to date there are no data in sporadic PD demonstrating that myocardial α-syn deposition precedes local NE deficiency.

### Implications and potential mechanisms of cardioselective sympathetic noradrenergic deficiency in PD

Our results demonstrating sympathetic noradrenergic deficiency in myocardium but not in scalp skin or SMG in PD fit with those from a recent organ survey of in vivo neuroimaging and post-mortem neurochemical data indicating that in Lewy body diseases, peripheral noradrenergic deficiency is organ-selective and is most prominent in the heart [21]. Understanding the bases for the unusual susceptibility of cardiac noradrenergic neurons might provide clues as to the greater vulnerability of nigrostriatal than of other dopaminergic neurons in the brain.

What are the potential bases for cardioselective catecholamine deficiency in PD? First, an obvious, unique characteristic of the heart is that it always is beating. This implies a uniquely high requirement for a continuous supply of energy. If there were a generalized impairment of mitochondrial functions, this could be manifested by a cardiac-specific decreased ability to store, release, or recycle catecholamines, as these are energy-requiring processes. Second, the heart stands out in terms of the extent of removal of circulating catecholamines by neuronal uptake mediated by the cell membrane NE transporter (NET). About 80% of tracer-labelled NE in arterial plasma is removed in one passage through the heart [24]. If there were a circulating toxic compound that could be transported into sympathetic nerves *via* the NET, there would be greater damage or a greater loss of sympathetic nerves in the heart than in other organs. Third, there is substantial DHPG production in the human heart. The concentration of DHPG in coronary sinus plasma is about twice that in the arterial plasma [25]. DHPG, the main intra-neuronal metabolite of NE, is produced by two enzymes acting in series, monoamine oxidase and aldehyde/aldose reductase. The immediate product of the first reaction is the catecholaldehyde 3,4-dihydroxyphenylglycolaldehyde (DOPEGAL). The high rate of DHPG production in the heart therefore implies a high rate of DOPEGAL production. DOPEGAL may exert autotoxic effects by inducing misfolding of intracellular proteins [26, 27]; if so, these effects would be particular noticeable in the heart.

### Greater loss of NE than of immunoreactive TH in PD myocardium

The quantitative immunofluorescence methodology used in the present study enabled the discovery that in PD patients, there is a more severe loss of myocardial NE (by 92% from control) than of immunoreactive TH (by 65% from control). Since immunoreactive TH is used as an index of catecholaminergic innervation, including in the heart [28], a potential explanation for the greater loss of NE than of immunoreactive TH could be the occurrence of a population of dysfunctional but still living residual sympathetic nerves [29]. Several functional abnormalities could work together to result in myocardial noradrenergic deficiency in Lewy body diseases, above and beyond effects of denervation alone [30]. Other potential explanations for greater loss of myocardial NE than of immunoreactive TH are persistence of non-functional TH epitopes in neuronal fragments, TH that has undergone oxidative damage due to reactive oxygen species or peroxynitrite [31], or TH that has been inactivated after forming quinoprotein adducts with catecholaldehydes [32]. In addition, misfolded α-syn might inhibit TH activity. Assessing the relative contributions of these factors is a matter for future research.

### Limitations

We included scalp skin and SMG, because they represent potentially biopsiable organs. Gastrointestinal tract would also be a biopsiable tissue, and according to a current concept, α-syn spreads in a prion-like manner from the gut to the brainstem *via* autonomic fibers [33]; however, gastrointestinal tract tissue autolyzes rapidly after death, and in the present study, post-mortem intervals up to 24 h were allowed.

It is possible that the observed differences in severity of the catecholaminergic lesion across the three compared organs can be explained by local differences in disease progression rather than by organ-specific vulnerability. Post-mortem studies such as ours cannot assess disease progression. Preliminarily, we have found that living PD patients with mild to moderate disease severity based on Uniform Parkinson Disease Rating Scale scores have (1) increased α-syn deposition in dermal sympathetic nerve fibers, (2) myocardial NE deficiency as indicated by ^18^F-DA-derived radioactivity, (3) normal immunoreactive TH in sympathetic noradrenergically innervated dermal constituents, and (4) normal NE content in skin tissue. This pattern of results, which is the same as in the present post-mortem study, suggests that increased α-syn deposition in dermal sympathetic nerves is not pathogenic throughout the course of PD.

The quality of the scalp skin samples was less than anticipated based on our experience with in vivo skin biopsies [11]. There are several potential explanations. One is post-mortem change, since published representative post-mortem images by another group using different methodology also did not show discrete nerve fibers [34]. Another possible explanation is that the autopsy samples in our study were frozen, whereas in vivo skin biopsies typically are fixed. The samples were from the scalp, and there are no published representative images of skin from this location. In addition, for most of the samples we had no control over the conditions of sampling, handling, and storage before the specimens were shipped to our laboratory.

For most of the samples from the PD patients, no clinical information was provided, only the neuropathologic diagnosis. All 3 PD patients who had been followed at the NIH had typical brainstem Lewy bodies and substantial putamen dopamine depletion. From the demographic information, the groups were quite elderly, and so generalizability to early-onset PD is unclear.

## Conclusions

The present results show that PD entails increased α-syn in scalp skin, SMG, and myocardium. The pathophysiological significance of increased intra-neuronal α-syn deposition seems to be organ-selective and prominent in the heart.

## Supplementary Information

**Additional file 1**. Demographic and individual data of control subjects (CTRL) and patients with Parkinson’s disease (PD). Individual values for immunoreactive signal intensities and neurochemical data from scalp skin, submandibular gland, and myocardium were tabulated. Mean values ± SEM are also shown. Other abbreviations: α-Syn = α-synuclein; Coloc. Index = α-synuclein-tyrosine hydroxylase colocalization index; cTnT = cardiac troponin T; DA = dopamine; DHPG = 3,4-dihydroxyphenylglycol; DOPAC = 3,4-dihydroxyphenylacetic acid; NE = norepinephrine; PD = Parkinson’s disease; PMI = post-mortem intervals; SMA = smooth muscle actin; SMG = submandibular gland; TH = tyrosine hydroxylase.

## Data Availability

The dataset supporting the conclusions of this article are anonymized individual data in Additional file 1.

## Abbreviations

cTnT: Cardiac troponin T
DA: Dopamine
DHPG: 3,4-Dihydroxyphenylglycol
DOPAC: 3,4-Dihydroxyphenylacetic acid
DOPEGAL: 3,4-Dihydroxyphenylglycolaldehyde
NE: Norepinephrine
PD: Parkinson’s disease
SMA: Alpha-smooth muscle actin
SMG: Submandibular gland
α-syn: α-Synuclein
TH: Tyrosine hydroxylase
